# Trichosporon infection in chronic kidney disease patients from a tertiary care hospital – a case series or an outbreak? An unanswered question but a well-managed problem

**DOI:** 10.3205/id000090

**Published:** 2024-11-11

**Authors:** Heera Hassan, Alina Nair L, Varsha N S, Jyothi R, Aravind Reghukumar, Sathyabhama M C, Ragi R G, Neethu Kishor, Mithu M G, Syed Ali, Kiran Gopal, Manjusree S, Swathi V Koramboor, Anuja U

**Affiliations:** 1 Department of Microbiology, Government Medical College, Thiruvananthapuram, India; 2 Department of Infectious Diseases, Government Medical College, Thiruvananthapuram, India; 3 Department of Community Medicine, Government Medical College, Thiruvananthapuram, India

**Keywords:** Trichosporon, chronic kidney disease, haemodialysis, magnesium, voriconazole

## Abstract

While the majority of *Trichosporon* spp. isolated in clinical laboratories are typically associated with episodes of colonization or superficial infections, this fungal species has gained recognition as an opportunistic pathogen, leading to invasive infections worldwide. In this article, we present a case series of *Trichosporon* spp. identified through conventional methods, complemented by MALDI-TOF analysis from a reference institute for a single sample. The reported cases occurred within a confined time frame, and the construction of an epidemic curve suggested a common source with intermittent exposure.

Despite the absence of identified breaches in infection prevention and control (IPC) in units with common exposure, this case series underscores the significance of considering Trichosporonosis in the differential diagnoses for post-transplant and chronic kidney disease patients, particularly those undergoing hemodialysis or utilizing Foley’s catheter. Notably, research gaps were identified, emphasizing the need for further exploration of factors such as the role of magnesium and prolonged antibiotic usage in the development of invasive *Trichosporon* infections and newer treatment modalities against biofilm producing yeast like fungi.

## Introduction

*Trichosporon* spp. are basidiomycetous yeast-like anamorphic organisms, found in tropical and temperate areas of the globe and are widely distributed in nature [1]. *Trichosporon* species can occasionally belong to the oral cavity, gastrointestinal tract and skin microbiota of humans [2]. They cause white piedra and onychomycosis in immunocompetent host as well as various localized and disseminated invasive infections in immunodeficient hosts particularly on treatment for hematological malignancies. There are only sporadic reports of urinary tract infections (UTIs) caused by *Trichosporon* *asahii* reported from India [3]. Although most *Trichosporon* spp. isolated in clinical laboratories are related to episodes of colonization or superficial infections, this fungus has been recognized as an opportunistic agent causing invasive infections in tertiary care hospitals worldwide [2]. At present, there are about 50 species of *Trichosporon* [4], of which about 16 species of *Trichosporon* are pathogenic [2]. Globally, *T. asahii* is the most common species causing invasive Trichosporonosis [2]. Risk factors for invasive infection are malignancy, indwelling vascular and urinary catheters, organ transplantation and broad-spectrum antibiotic therapy [5]. Virulence factors for Trichosporonosis include glucuronoxylomannan in cell wall, proteases, phospholipases, and the ability to form biofilms. They form true mycelia, blastoconidia, and arthroconidia. Virulence factors and morphological structures may be exhibited differently in different species. Their ubiquity and biofilm formation may create confusion between colonized and truly infected patients. These infections present a challenge for clinicians and microbiologists, as there are no clear and specific guidelines for the reporting and interpretation of *Trichosporon* spp. isolated from clinical specimen [2].

We report 5 cases with *Trichosporon* *asahii* in chronic kidney disease (CKD) patients in our tertiary care hospital.

## Case descriptions

### Case 1

A 42-year-old man underwent a renal transplant on February 5, 2023, for end-stage renal disease (ESRD) due to focal segmental glomerulosclerosis from a donor with documented fungal infection. The patient was catheterised in the OT. On postoperative day 42 (31^st^ day of indwelling catheter insertion), he developed cellulitis and deep vein thrombosis (DVT) in his left lower limb along with signs of sepsis. Although the blood cultures were sterile, direct microscopy of urine obtained from the catheter showed 10–12 pus cells per high power field (HPF) with septate hyphae and arthroconidia on the wet film. Further culture of the urine revealed more than 10^5^ CFU/ml of chalky white, dry and wrinkled colonies in blood agar and MacConkey agar. The same colonies were consistently isolated from urine specimen taken three times (the last sample was collected soon after a catheter change), as well as from pus aspirates from a cellulitis-affected limb. These samples were processed in our laboratory on multiple occasions during March and April 2023. After preliminary identification as *Trichosporon*, the isolate was sent to a reference centre which confirmed the identification as *Trichosporon* *asahii* by MALDI-TOF. Antifungal susceptibility test results were also provided by the reference centre. The absence of breakpoints in Clinical & Laboratory Standards Institute (CLSI) guidelines for *Trichosporon* *asahii* was one of the hurdles in choosing an appropriate antifungal treatment. Since the patient did not show improvement on intravenous Fluconazole 800 mg loading followed by 200 mg OD, therapy was switched to oral Voriconazole 200 mg twice a day. In view of graft dysfunction with acute kidney injury, maintenance haemodialysis (MHD) was initiated, which were done at transplant intensive care unit (ICU) and haemodialysis (HD) room. Despite these efforts, the patient expired on the 65^th^ postoperative day on April 2023.

### Case 2

A 50-year-old male presented to nephrology outpatient department (OPD) with reduced urine output, generalised oedema and excessive fatigability. He was diagnosed to have right sided pyelonephritis, acute on chronic kidney disease, and systemic arterial hypertension. He was admitted in the second week of May and was initiated on MHD on alternative days throughout the month of May 2023 from HD room. Due to persistent fever and elevated leukocyte counts, a urine sample was collected from a catheter that had been in place for 10 days. Direct microscopy showed 10–12 pus cells per high power field (HPF) with septate hyphae and arthroconidia. Urine culture yielded chalky white, dry and wrinkled colonies in blood agar and MacConkey agar on three occasions in May 2023, indicating an ongoing catheter-associated urinary tract infection (CAUTI). The isolate was identified as *Trichosporon* spp. in our laboratory. The blood cultures were sterile. He was started on Amphotericin B (AmB) deoxycholate 50 mg daily and intravenous Fluconazole 200 mg OD after a loading dose of 800 mg. He was discharged on clinical improvement and after his urine culture became sterile.

### Case 3

A 78-year-old male with a history of coronary artery disease, systemic arterial hypertension, ischemic dilated cardiomyopathy, atypical haemolytic uremic syndrome, CKD on MHD was admitted in the last week of June 2023 and was continued on MHD until the first week of July 2023. The initial urine sample from indwelling catheter was sent for culture due to a fever spike. Urine direct microscopy showed 8–10 pus cells/HPF with septate hyphae and arthroconidia. Urine culture yielded 50,000–70,000 CFU/ml of chalky white, dry and wrinkled colonies in blood agar and MacConkey agar. The same results repeated thrice in the last week of June, with sterile reports in blood culture. The isolate was identified as *Trichosporon* spp. in our laboratory. He was started on Amphotericin B deoxycholate 50 mg once a day and intravenous Fluconazole 200 mg OD after a loading dose of 800 mg, and discharged after becoming culture negative and better.

### Case 4

A 16-year-old male diagnosed with CKD, right atrophied kidney, hypertension and anaemia of CKD, while on MHD on OP basis from first week of June 2023 for every 3 days per week presented with fever and chills in the second week of July. On account of the elevated total leucocyte counts (total counts: 16,000 cells/µL), blood culture was sent from central and peripheral lines. Both the samples yielded pure growth of chalky white, dry and wrinkled colonies in blood agar and MacConkey agar, identified as *Trichosporon*. He was diagnosed to have central line related blood stream infection and was initiated on intravenous Voriconazole 6 mg/kg Q12H for one day followed by 4 mg/kg Q12H, and discharged after becoming culture negative and clinically better. His central venous catheter was also revised.

### Case 5

A 46-year-old male with known CKD–ESRD underwent diseased donor renal transplant on February 5, 2023, from the same donor as that of the first case. Later he was admitted for obstructive uropathy post-ureteric re-implantation. He was admitted on July 21, 2023, and was initiated MHD. In the first week of August 2023, due to persistent fever spikes, urine from the indwelling catheter and blood samples were sent for culture. Urine direct microscopy showed 8–10 pus cells/HPF with septate hyphae and arthroconidia. Urine culture yielded more than 10^5^ CFU/ml of chalky white, dry and wrinkled colonies in blood agar and MacConkey agar. The blood cultures were sterile. The isolate was identified as *Trichosporon* spp. in our laboratory. Blood culture was sterile. He was initiated on liposomal Amphotericin B 5 mg/kg once a day and oral Voriconazole 6 mg/kg Q12H for one day followed by 4 mg/kg Q12H, but expired due to other co-existing morbidities.

## Laboratory diagnosis

**Figure 1:**
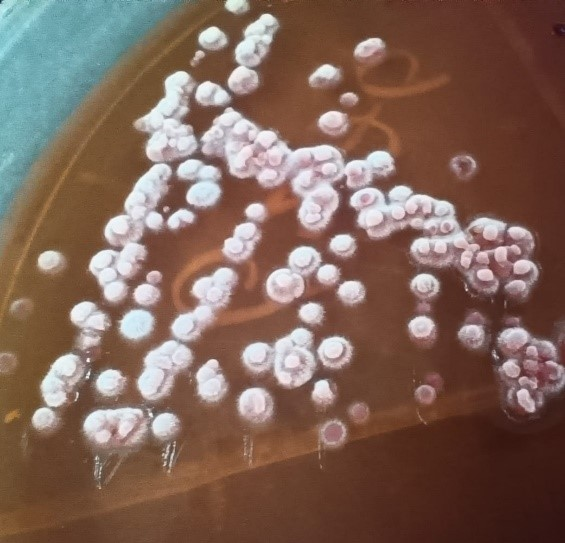
Dry wrinkled colonies of *Trichosporon* on MacConkey agar incubated at 37°C after 24 hours of incubation

**Figure 2:**
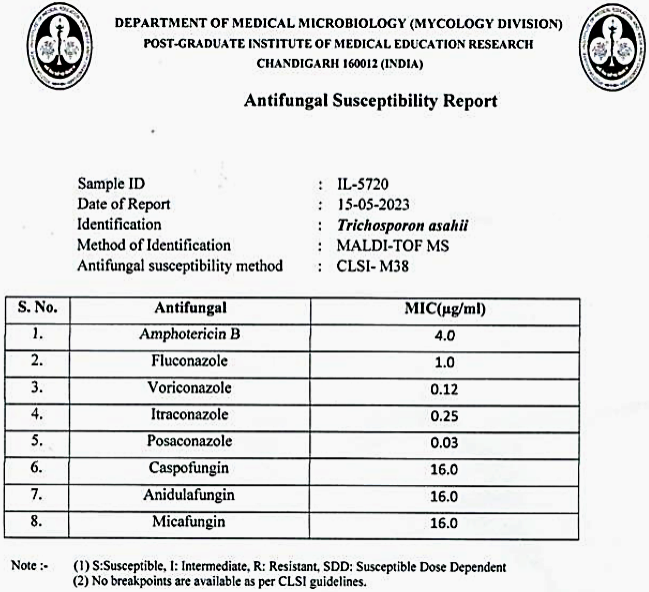
Report of MALDI-TOF

The specimen was inoculated in blood and MacConkey agar and incubated at 37°C. At the end of 24 hours the colonies were chalky white, dry and wrinkled in blood agar and MacConkey agar (Figure 1 (Fig. 1)). On gram staining septate hyphae with arthrospores and few budding yeast cells were seen. Urea hydrolysis was performed by streaking the colony on Christensen’s urea agar to differentiate it from *Geotrichum*. Urea was hydrolysed after 24 hours of incubation. In all 5 cases including blood sample of the 4^th^ patient, the isolate was identified as *Trichosporon* species through its cultural characteristics and urea hydrolysis. In the first case, identification was confirmed by Vitek MALDI-TOF MS (bioMérieux) using the Vitek MS knowledge base version 3.0 at PGIMER, Chandigarh (a national referral centre for Research on Fungi of Medical Importance). Antifungal susceptibility testing was also performed at this reference laboratory (Figure 2 (Fig. 2)).

## Discussion

Trichosporonosis is an emerging fungal infection mainly caused by *Trichosporon* *asahii*. UTI due to *Trichosporon* has rarely been reported. Majority of the *T. asahii* infections reported in literature were in neonates or immunocompromised patients especially with haematological malignancies. The major risk factors identified in various studies were prior antibiotic use, invasive medical equipment and chemotherapy. The rising utilization of various medical devices, combined with the capability to form biofilms, promotes the proliferation of *Trichosporon* spp. and its involvement in device-associated infections. 

In this case series, all the patients affected were males, contrary to previous studies on *Trichosporon* infection where males constituted only 65% of the total cases [3], [6]. The mean age of affected population was found to be 46.4 years. Four out of five patients had UTI and one patient had catheter-related bloodstream infection (CRBSI) due to *Trichosporon*. Four out of five patients were on Foley’s catheter at time of diagnosis with mean duration of catheter use being 13.5 days, while the mean duration in a previous study [3] was 18.8 days.

All the patients in our report were immunocompromised as they were CKD patients and two of them were on post-renal transplant immunosuppression. A search for other contributory risk factors from previous studies showed that in our study 60% were hypertensive, none were diabetic, 40% were anaemic, and 40% were on broad spectrum antibiotics. All patients in our study had undergone haemodialysis through central venous catheter in right internal jugular vein (IJV) and 80% had Foley’s catheter. As all patients shared treatment modalities like haemodialysis and as majority had Foley’s catheterisation, the possibility of an outbreak was considered (Table 1 (Tab. 1)). Moreover, by definition as *Trichosporon* infections are rare, even a single case should be flagged as an outbreak and investigated.

**Figure 3:**
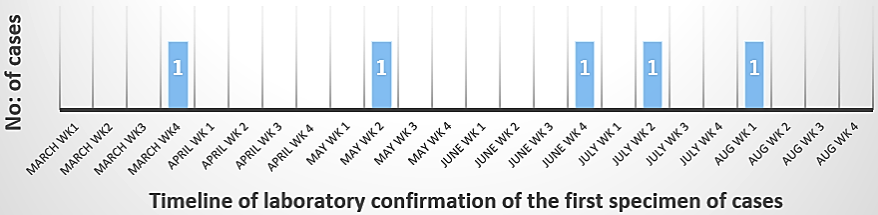
Advanced epidemic curve: distribution of laboratory confirmation of cases over time

An advanced epidemic curve [7] was created as a part of outbreak investigation (Figure 3 (Fig. 3)).The bar graph thus charted hinted a common source intermittent exposure type of outbreak [8], which led us to focus on MHD in the same HD room. The index patient had extensive lower limb cellulitis due to *Trichosporon* *asahii* (confirmed by MALDITOF), and he had been shifted to haemodialysis room for haemodialysis. All individuals had exposure to the HD room, and it was hypothesized that breaches in central line and urinary catheter insertion/maintenance might have contributed to CRBSI and UTIs caused by *Trichosporon*. Due to various constraints, confirmation of species by MALDITOF was possible only in the index case. Despite focussed investigation in HD room, no identifiable breaches in central line or Foley’s catheter insertion or maintenance bundle were detected to prove the hypothesis. Extensive environmental sampling was also conducted. The plastic rod used for mixing the solution as well as the racks in washing room, joining ports of dialysis machines and storage racks of reused dialysis tubes were covered with salt like deposits. Environmental sampling of HD room included swabs from salt like deposits and dialysis fluids. The culture of the environmental samples yielded mixed growth of bacteria as well as fungus including *Aspergillus* spp., but failed to culture *Trichosporon*, despite diligent efforts.

After this, fumigation and thorough surface cleaning was carried out in the HD room following which environmental surveillance culture swabs became sterile. The supervising staff were directed asked to monitor the reconstitution of dialysate solution. Sensitization sessions were conducted to augment IPC practices in HD room especially periodic cleaning of high touch areas and bundle care approaches with regard to CVC and Foley’s catheter. Laboratory based and clinical surveillance was strengthened to detect further *Trichosporon* infections. After strengthening of IPC measures in dialysis room, further cases of *Trichosporon* infection have not been reported over next 6 months which indirectly suggests that the postulated hypothesis was probably correct even though it could not be proved.

A literature search was done to find a possible explanation as for why *Trichosporon* infection from index case occurred on only few patients among hundreds of patients undergoing dialysis in a dialysis room led us to consider the role of magnesium in *Trichosporon* virulence. Magnesium (Mg) haemostasis gets jeopardised in chronic CKD patients with creatinine clearance <10 ml/min and with variations in magnesium concentrations in dialysate fluids [9], [10]. Following the institution of chronic haemodialysis in patients with non-functioning kidney, the major determinant of magnesium balance is the concentration of magnesium in the dialysate [10]. Besides, in-vivo study results show that an increase in Mg, accelerated hyphal growth in *T. asahii* [11]. Considering the postulate, the levels of magnesium in dialysate fluids and the methods of reconstitution were supervised. The constitution of dialysis fluids was according to manufacturer’s instructions. 

Fungal biofilm state is the main cause of treatment failure and recurrence of fungal infections. Azoles have only moderate efficacy against candida biofilms [12]. This could be extrapolated to biofilms of *Trichosporon* and may be the reason for the failure of prophylactic Fluconazole therapy. Out of all the antifungal drugs used clinically, only echinocandins and liposomal formulations of AmB can be used to treat biofilm-based infections of yeast [12]. Nephrotoxicity can be reduced but not eliminated by the liposomal formulation of AmB, which restricts its use in CKD patients [12]. This along with the intrinsic resistance of *Trichosporon* to echinocandins makes the therapeutic management challenging [13]. The role of novel non-antifungal potentiators along with anti-fungal drugs against biofilms needs further research in the management of such patients as well as for stewardship activities.

## Study limitations

The data collection encountered challenges due to its retrospective nature, leading to difficulties in accessing crucial clinical data. Data on serum magnesium levels, and magnesium levels in dialysate fluids was unavailable. The study could not identify the presence of *Trichosporon* infection or colonization in the hairs and nails of individual patients or healthcare workers, potentially contributing to invasive infections in the context of magnesium imbalance. Genomic sequencing was not performed on *Trichosporon* isolates to prove common source intermittent exposure outbreak. Isolation of *Trichosporon* was not possible from the environmental samples. Given the inability to pinpoint a common source for the outbreak and reliance on assumptions, the above cases may be considered as a case series.

## Conclusions

*Trichosporon* *asahii* is an emerging fungal pathogen which can lead to serious infections in immunocompromised. This case series highlight the importance of considering Trichosporonosis also as one of the differential diagnoses in post-transplant and chronic kidney disease patients especially if they are on hemodialysis or Foley’s catheter. The role of magnesium in promoting virulence and transition from yeast to hyphal forms of *Trichosporon* needs to be explored further. In case of outbreaks of *Trichosporon* infection in dialysis units, assessing magnesium levels in dialysate solution with combination therapy targeting biofilms should also be considered along with augmentation of IPC practices including central line and Foley’s catheter insertion and maintenance bundles. 

## Notes

### Acknowledgement

We would like to express our gratitude to Dr Anuja M, former Associate Professor of Health Education, State PEID Cell and our infection control nurses Deepa and Salini for their help in data collection and infection prevention and control activities. We are grateful to PGIMER, Chandigarh for the prompt MALDI-TOF identification and antifungal susceptibility report.

### Competing interests

The authors declare that they have no competing interests.

## Figures and Tables

**Table 1:**
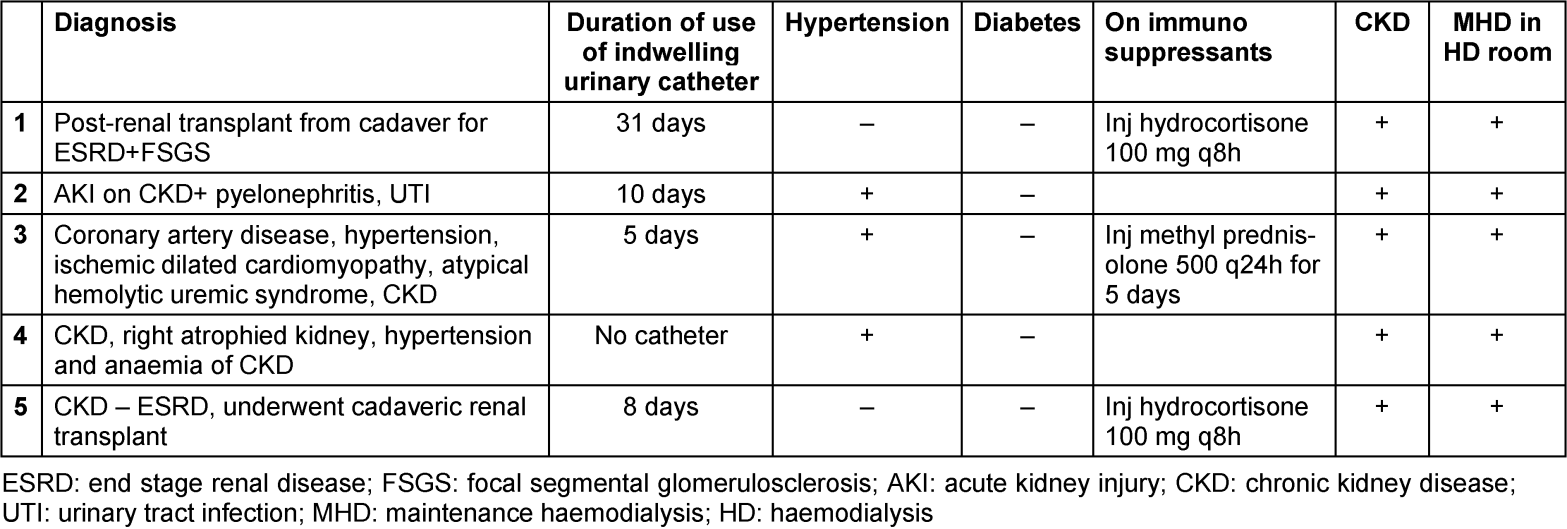
Patient risk factors
